# Impact of N‐Doping on MoSe_2_ Monolayer for PH_3_, C_2_N_2,_ and HN_3_ Gas Sensing: A DFT Study

**DOI:** 10.1002/open.202400210

**Published:** 2024-11-21

**Authors:** Mim Khatun, Mahabub Hasan Rocky, Abdullah Al Roman, Debashis Roy, Md. Alamgir Badsha, Mohammad Tanvir Ahmed

**Affiliations:** ^1^ Department of Physics Jashore University of Science and Technology Jashore 7408 Bangladesh

**Keywords:** Adsorption, DFT, Gas sensor, MoSe_2_, N-doping

## Abstract

In this research, the different characteristics of MoSe_2_ and N‐doped MoSe_2_ monolayers were studied using density functional theory calculations. The negative cohesive energy (−5.216 eV for MoSe_2_ and −5.333 eV for N‐MoSe_2_) verified their energetical stability. The variation of structural, electronic, and optical properties of MoSe_2_ and N‐MoSe_2_ via adsorption of PH_3_, C_2_N_2_, and HN_3_ gases are studied. The N‐doping results in a stronger adsorbent‐gas interaction, resulting in maximum adsorption energy of −0.036, −0.033, and −0.198 eV for the selected gases. The MoSe_2_ and N‐MoSe_2_ monolayers showed a direct band gap of 1.48 eV and 1.09 eV, respectively. However, upon interaction with the gases, a notable shift in the band gap of both adsorbents is observed. N‐MoSe_2_ showed semiconductor‐to‐conductor transition via C_2_N_2_ and HN_3_ adsorption. The sensitivity of MoSe_2_ for the selected gases has improved remarkably via N‐doping. Also, HN_3_ gas can be easily detected by the N‐MoSe_2_ monolayer due to the greater changes in work function (0.45 eV). The absorption coefficient of both adsorbents is over 10^5^ cm^−1^ order in the UV region, which suffers a mild peak shifting due to gas adsorption. This study suggests that N‐MoSe_2_ can be a potential candidate for selected gas sensing.

## Graphical abstract

This study uses DFT calculations to explore the stability, electronic, and optical properties of MoSe_2_ and N‐doped MoSe_2_ monolayers, highlighting their enhanced gas sensing potential after N‐doping

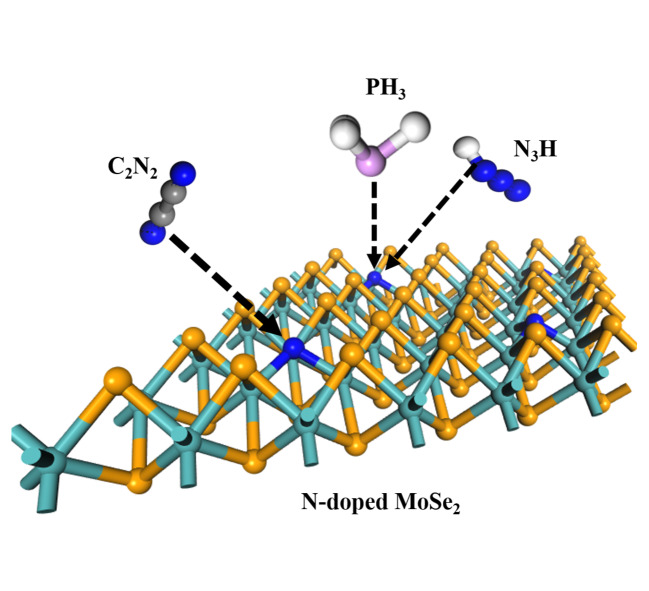

## 1 Introduction

In recent times, toxic gases have increased day by day in our environment from different sources. Different types of dangerous gases such as PH_3_, C_2_N_2_, H_3_N, CO, CO_2_, SO_2,_ NO, NH_3_, O_3_ etc. holds in our environment. The poisonous gases are created by power plants, motorized traffic, biological waste, industry, chemical laboratory and so on.^[1]^ So, detecting and monitoring these toxic gases plays a pivotal role in protecting our health and providing a better living environment. Phosphine (PH_3_) is a colorless, flammable, highly toxic compound. It appears to be mainly a redox toxin, causing cell damage by including oxidative stress. It can damage the kidney, cause pulmonary edema, abdominal pain, and respiratory systems etc.[^1^, ^2^] Cyanogen (C_2_N_2_) is a colorless and highly toxic gas with a pungent odor. It is flammable, forms cyanide in the body damages the respiratory system and is irritant to the eyes.[^3^, ^4^] Hydrogen azide (HN_3_), also known as hydrazoic acid or azoimide is a highly toxic, colorless, pungent odor, volatile, and explosive liquid at room temperature and pressure. It becomes a very dangerous explosive and damages our respiratory systems, multi‐organ failure, and irritation to the eyes, nose, throat, skin etc.[^5^, ^6^]

Nowadays, researchers are interested in two‐dimensional (2D) nanomaterials due to their layered structures and enhanced properties.[^7^, ^8^, ^9^] Transition metal dichalcogenide (TMD) monolayers are semiconductors of the type MX_2_, with M a transition metal atom (Ti, Mo, W, Ta, Nb etc.) and X a chalcogen atom (O, S, Se, or Te).^[10]^ A single layer of M atoms is sandwiched between two layers of X atoms. TMDs atomic‐layer of dichalcogenides nanostructures such as MoSe_2_, MoS_2_, WSe_2_, WS_2_, WSSe, SnS_2_, etc. can exhibit outstanding electrical properties and be utilized in various potential applications.[^11^, ^12^] Nayeri *et al*. observed the transport and optical properties of MoS_2_, MoSe_2_, WS_2_ and WSe_2_ semiconducting TMDs in the presence of the NH_3_, NO, NO_2_, and O_2_ gases to assess their potentials as gas sensors and the materials lead to significant changes in their transmission spectrum.^[13]^ Late *et al*. reported that the mono‐layer MoSe_2_ is a potential NH_3_ gas sensor.^[14]^ Due to the excellent electronic, transport and optical properties, and sensing capabilities of MoSe_2_, we selected the MoSe_2_ monolayer for our present work.

According to the investigation of Zhang et al., Pd and Pt‐decorated MoSe_2_ hierarchical nanoflowers demonstrated a high sensitivity, stability, and response rate to SO_2_ gas.^[15]^ Liu *et al*. reported Al‐doped MoSe_2_ as a promising biosensor and Al‐doped MoSe_2_ behaves as an electron donor which significantly enhances the electrical conductivity of the monolayer.^[16]^ Ayesh investigated the adsorption of H_2_S and SO_2_ gases on Zn‐modified MoSe_2_ and proposed that Zn‐modified MoSe_2_ increased the H_2_S gas sensing ability.^[17]^ Lin et al. showed that metal decoration can significantly enhance the sensitivity of MoSe_2_ for NO_2_ gas.^[18]^ Li *et al*. experimentally and theoretically analyzed the flower‐like intrinsic and Pt‐doped MoSe_2_ gas‐sensitive materials for the detection of NO_2_ gas.^[19]^ According to Choi et al., Nb doping on MoSe_2_ improved NO_2_ gas sensing stability.^[20]^ Zhang *et al*. showed that the adsorption of H_2_ and C_2_H_2_ on Rh‐MoSe_2_ increases their energy band gap.^[21]^ After examining the different atoms doping on MoSe_2_, we decided to dope an atom on the monolayer of MoSe_2_, which had not been done before. Distinct studies suggest that the addition of nitrogen (N) atoms can enhance gas‐sensing capability.[^22^, ^23^] CongCong *et al*. reported N‐doped graphene as a potential candidate for CO gas sensing from air.^[22]^ Abbasi *et al*. investigated the adsorption behaviors of NH_3_ on N‐doped TiO_2_ anatase nanoparticles and showed that N‐doped nanoparticles have higher sensing capability than pristine ones.^[23]^ The charge transfer property of the N atom and enhanced sensing of gas adsorption after doping the N atom in nanosheets, encourage our doping N atom in our present work.

In this paper, we performed density functional theory (DFT) computations to study the gas sensing capability of pristine MoSe_2_ and N‐doped MoSe_2_ (N‐MoSe_2_) monolayers toward PH_3_, C_2_N_2_ and HN_3_ toxic gases. The change of structural, electronic, and optical properties because of N doping on the MoSe_2_ monolayer as well as due to the adsorption of PH_3_, C_2_N_2_ and HN_3_ molecules on the designed monolayer are thoroughly studied. Ultimately, similar investigations regarding N‐MoSe_2_ have not been disclosed previously.

## 2 Calculating Method

The impact of PH_3_, C_2_N_2_, and HN_3_ gas molecules on both pristine and N‐doped MoSe_2_ monolayers was investigated by DFT study. All the simulations for structural, electronic and optical property analysis were outdone by the Cambridge Serial Total Energy Package (CASTEP) module of Materials Studio software.^[24]^ The relaxed pristine and complex geometries were obtained via the Brayden‐Fletcher‐Goldfarb‐Shanno (BFGS) method.^[25]^ The geometrical, electronic, and optical properties were analyzed using the generalized gradient approximations (GGA) which involve Perdew Burke Ernzerhof (PBE) calculations to define the electron exchange and correlation.^[26]^ For geometry optimization, the convergence criteria for energy, maximum force, maximum stress, and maximum displacement were set to 10^−5^ eV/atom, 0.03 eV/Å, 0.05 GPa, and 0.001 Å, respectively. Throughout all the simulations a vacuum space of 20 Å was employed along the z‐direction to avoid the interlayer interaction.^[27]^ We have constructed a 3×2×1 supercell of MoSe_2_ monolayer. For the overall calculation, we used 450 eV cutoff energy with 3×3×1 k‐point sampling.^[28]^ The efficiency of the calculations was improved by maintaining the self‐consistent field (SCF) tolerance to 10^−6^ eV/Atom.^[29]^ An ultrasoft pseudopotential was employed to process the inner core electrons resulting in a reduced computational cost.

## 3 Results and Discussion

### 3.1 Geometry Optimization

**Figure 1:**
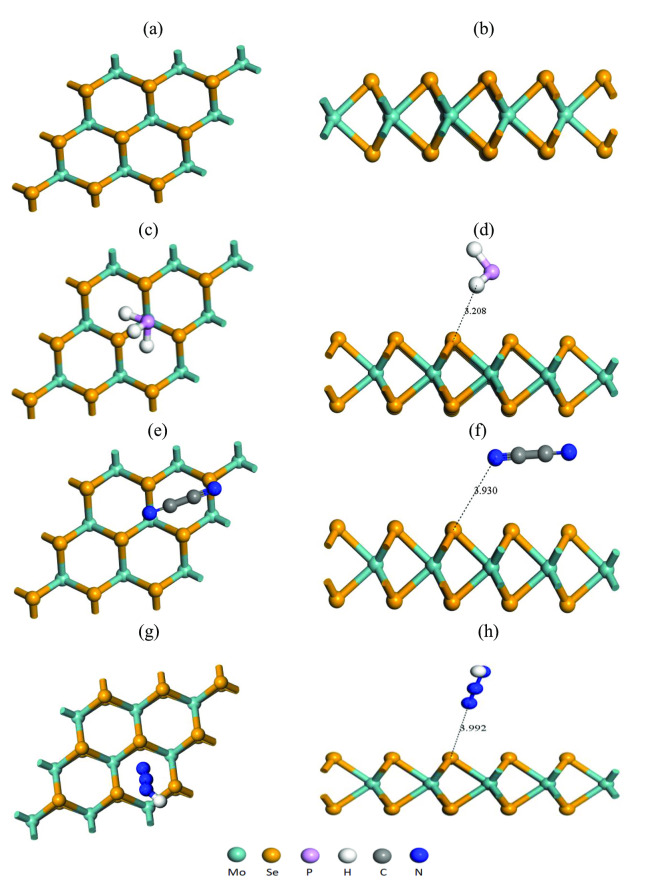
Optimized structures of (a) front and (b) edge view of pristine MoSe_2_ monolayer; (c) front and (d) edge view of MoSe_2_ with PH_3_; (e) front and (f) edge view of MoSe_2_ with C_2_N_2_; (g) front and (h) edge view of MoSe_2_ with HN_3_ gases.

**Figure 2:**
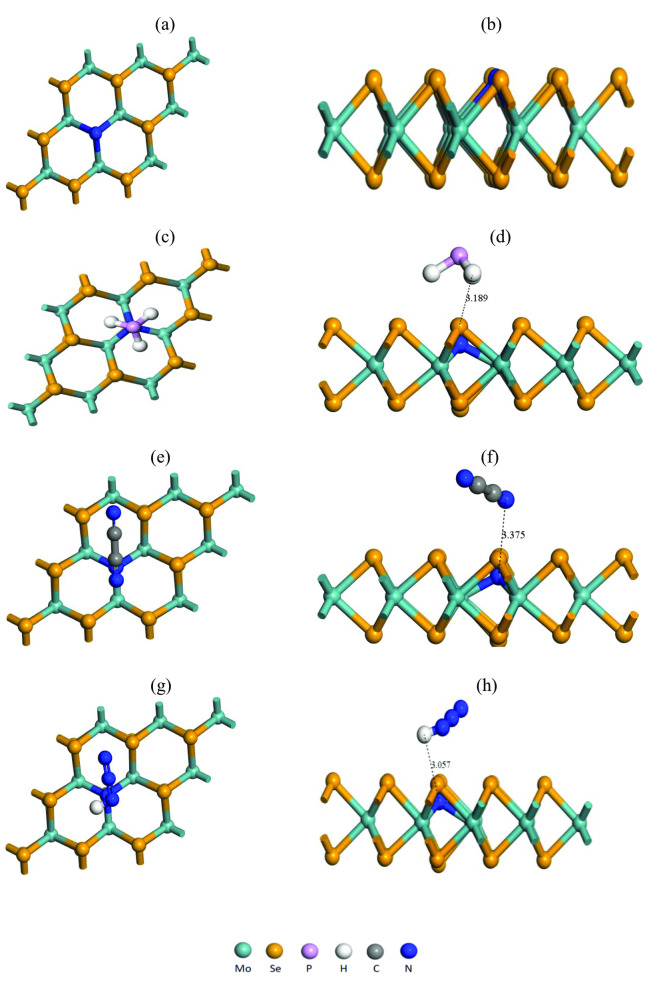
Optimized structures of (a) front and (b) edge view of N‐MoSe_2_ monolayer; (c) front and (d) edge view of N‐MoSe_2_ with PH_3_; (e) front and (f) edge view of N‐MoSe_2_ with C_2_N_2_; (g) front and (h) edge view of N‐MoSe_2_ with HN_3_ gases.

One of the important properties of any material is structural stability. In our present work, we studied the geometrical structure of pristine MoSe_2_ and N‐MoSe_2_ monolayers (prior to and subsequent to the adsorption of selective toxic gases) are represented in Figures 1 and 2, respectively. We have calculated cohesive energy from the following equation,^[30]^

(1) $$E_{cohesive}=\frac{1}{n}\;\left[E_{\left(monolayer\right)}-X_{\left(Mo\right)}E_{\left(Mo\right)}-Y_{\left(Se\right)}E_{\left(Se\right)}-Z_{\left(N\right)\;}E_{\left(N\right)\;}\right]\;$$

here, n is the total number of atoms in the monolayer, X_(Mo)_, Y_(Se),_ and Z_(N)_ are the total number of Mo, Se, and N atoms. E_(monolayer)_, and *E*_(Mo)_, *E*_*(*Se),_ and *E*_(N)_ are the energy of the adsorbent, the energy of isolated Mo, Se, and N atoms respectively. We found the cohesive energy from the following equation, the pristine MoSe_2_ monolayer is −5.216 eV and the N‐MoSe_2_ monolayer is −5.333 eV. So, those two structures are stable and possible to synthesize. Analyzing cohesive energy, the N‐MoSe_2_ is more stable than the pristine MoSe_2_.

We have studied the optimized structures of MoSe_2_ and N‐MoSe_2_ monolayers in the presence of various toxic gases. The optimized structures of the MoSe_2_ and N‐MoSe_2_ along with adsorbed selective toxic gases PH_3_, C_2_N_2_, and HN_3_ are shown in Figures 1 and 2, respectively. The optimized structure of both pristine MoSe_2_ and N‐MoSe_2_ demonstrates that the complex structures are slightly deformed from the pristine structure. The geometrical bond lengths between various atoms of the optimized structure of selective toxic gases, MoSe_2_, and N‐MoSe_2_ are represented in Tables 1, 2, 3, respectively. According to the obtained result, all bond lengths increased slightly in MoSe_2_ and N‐MoSe_2_ after gas absorption which caused very negligible deformation in structure due to gas adsorption. In single layer MoSe_2_ structure, the optimized bond length of Mo−Se is 2.515 Å which satisfies previous reports[^31^, ^32^] and N‐MoSe_2_ monolayer, the optimized bond length of Mo−Se is 2.525 Å, which is slightly varied due to N doping.

**Table 1:** Average bond length (Å) for isolated gas molecules.

Bond	PH_3_	C_2_N_2_	HN_3_
H−P	1.425	–	–
C−N	–	1.866	–
H−N	–	–	1.464
N−N	–	–	1.627
C−C	–	1.367	–

**Table 2:** Average bond length (Å) changes for gas adsorption on MoSe_2_ monolayer.

Bond	MoSe_2_	MoSe_2_+PH_3_	MoSe_2_+C_2_N_2_	MoSe_2_+HN_3_
Se−Mo	2.515	2.516	2.520	2.520
H−P	–	1.424	–	–
C−N	–	–	1.867	–
H−N	–	–	–	1.464
C−C	–	–	1.367	–
N−N	–	–	–	1.627

**Table 3:** Average bond length (Å) changes for gas adsorption on N‐MoSe_2_ monolayer.

Bond	N‐MoSe_2_	N‐MoSe_2_+PH_3_	N‐MoSe_2_+C_2_N_2_	N‐MoSe_2_+HN_3_
Se−Mo	2.525	2.525	2.553	2.523
N−Se	2.774	2.771	2.768	2.761
H−P	–	1.426	–	–
C−N	–	–	1.866	
H−N	–	–	–	1.771
N−Mo	1.993	1.993	1.993	2.008
C−C	–	–	1.366	–
N−N	–	–	–	1.627

### 3.2 Adsorption Energy Analysis

The adsorption energies associated with the adsorption distances are evaluated and shown in Table 4 for selective toxic gas molecules. We have calculated the adsorption energies ($E_{Ads\;}$ ) for the PH_3_, C_2_N_2_ and HN_3_ gas molecules on the monolayer by the following equation,^[30]^

(2) $$E_{Ads\;}=E_{monolayer+gas\;molecule}\;-E_{monolayer}-E_{gas\;molecules}\;$$

**Table 4:** Adsorption energy and adsorption distance of MoSe_2_ and N‐MoSe_2_ for selective gases.

Adsorbed gas	Adsorption energy (eV)		Adsorption distance (Å)	
	MoSe_2_	N‐doped MoSe_2_	MoSe_2_	N‐doped MoSe_2_
PH_3_	0.013	−0.036	3.208	3.189
C_2_N_2_	−0.018	−0.033	3.930	3.375
HN_3_	−0.029	−0.198	3.992	3.057

where, $E_{monolayer+gas\;molecules}$ ,$\;E_{monolayer}$ and $E_{gas\;molecules}$ are the energy of the complex (after gas adsorption), the energy of the MoSe_2_ (or N‐MoSe_2_) monolayer and the energy of the isolated gas molecules.

In the MoSe_2_ monolayer, the adsorption energies are negative for the selective toxic gas molecules except for PH_3_ gas molecules. Hence, the gas molecules C_2_N_2_ and HN_3_ show attraction with MoSe_2_. In the N‐MoSe_2_ monolayer, each of the gas molecules PH_3_, C_2_N_2_, and HN_3_ are adsorbed comparatively stronger than MoSe_2_, among which HN_3_ is the most strongly adsorbed gas molecule. The adsorption distances are small for the adsorbent N‐MoSe_2_ but larger for MoSe_2_.The small adsorption distance and higher adsorption energy for the adsorbent N‐MoSe_2_ mean that the N‐MoSe_2_ has a higher gas sensing ability for the selected toxic gases than the MoSe_2_. The N‐MoSe_2_ monolayer showed stronger adsorption for PH_3_ and HN_3_ gases compared to boron nitride (BN) nanosheet, and BN nanotube.[^1^, ^33^] However, the adsorption of C_2_N_2_ on N‐MoSe_2_ is comparatively weaker than previously studied adsorbents.[^4^, ^34^] All the gases show physisorption on MoSe_2_ and N‐MoSe_2_ monolayers.

The recovery time (RT) is a very vital property for developing a gas sensor. We have calculated the RT from the Equation 3,^[30]^

(3) $$\tau =\;\frac{1}{f_{o}}\;\;e^{\frac{-E_{Ads}\;}{KT}\;}\;$$

where, K and T represent the Boltzmann's constant (8.62× 10^−5^ eV.K^−1^) and temperature. Experimentally, a sensor is recovered by exposing it to UV light with a frequency of ($f_{o}$ =10^12^ to 3× 10^14^ Hz).^[35]^ In this study, we used $f_{o}$ =10^12^ Hz and T=298 K. From Table 5, the RTs of MoSe_2_ and N‐MoSe_2_ from the adsorbed gas molecules are found between picoseconds (ps) and nanoseconds (ns) range. RTs of the pristine MoSe_2_ are lower than N‐MoSe_2_ due to the higher adsorption energies of the N‐MoSe_2_. All the adsorbent reveals a fast recovery from the adsorbed gases which is preferable for sensing applications.

**Table 5:** Recovery times of the monolayers from the selective toxic gases.

Gas molecules	MoSe_2_	N‐MoSe_2_
PH_3_	0.60 ps	4.06 ps
C_2_N_2_	2.02 ps	3.61 ps
HN_3_	3.09 ps	2.23 ns

### 3.3 Electronic Properties Analysis

#### 3.3.1 Hirshfeld Charges

The difference between the molecular and unrelaxed atomic charge densities is known as the Hirschfeld charges, which are charges related to the deformation density.^[36]^ Table 6 shows the average Hirshfeld charges of pure gases before adsorption whereas Tables 7 and 8 show the average Hirshfeld charges of elements of MoSe_2_ and N‐MoSe_2_ monolayers before and after adsorption, respectively. All the elements in the MoSe_2_ almost remain the same in average Hirshfeld charge after gas adsorption. In the MoSe_2_, the Mo atoms are partially positively charged whereas the Se atoms are partially negatively charged due to their higher electronegativity. The average Hirshfeld charges of the elements in the N‐MoSe_2_ slightly change after gas adsorption. The Se and N atoms revealed partially negative charges because of their high electronegativity, i. e. bond pair electrons (BPE) are attracted toward the Se and N atoms.

**Table 6:** Average Hirshfeld charges of pure gases before adsorption.

Elements	PH_3_	C_2_N_2_	HN_3_
P	0.04	–	–
H	−0.01	–	0.14
C	–	0.11	–
N	–	−0.11	−0.04

**Table 7:** Average Hirshfeld charges of pristine and gas‐adsorbed MoSe_2_.

Elements	MoSe_2_	MoSe_2_+PH_3_	MoSe_2_+C_2_N_2_	MoSe_2_+HN_3_
Mo	0.17	0.17	0.17	0.17
Se	−0.08	−0.08	−0.08	−0.08
P	–	0.03	–	–
H	–	−0.25	–	0.14
C	–	–	0.10	–
N	–	–	−0.11	−0.05

**Table 8:** Average Hirshfeld charges of pristine and gas‐adsorbed N‐MoSe_2_.

Elements	N‐MoSe_2_	N‐MoSe_2_+PH_3_	N‐MoSe_2_+C_2_N_2_	N‐MoSe_2_+HN_3_
Mo	0.19	0.19	0.19	0.19
Se	−0.08	−0.08	−0.08	−0.07
N	−0.33	−0.33	−0.18	−0.12
P	–	0.03	–	–
H	–	−0.03	–	0.09
C	–	–	0.10	–

#### 3.3.2 Electron Density Difference

**Figure 3:**
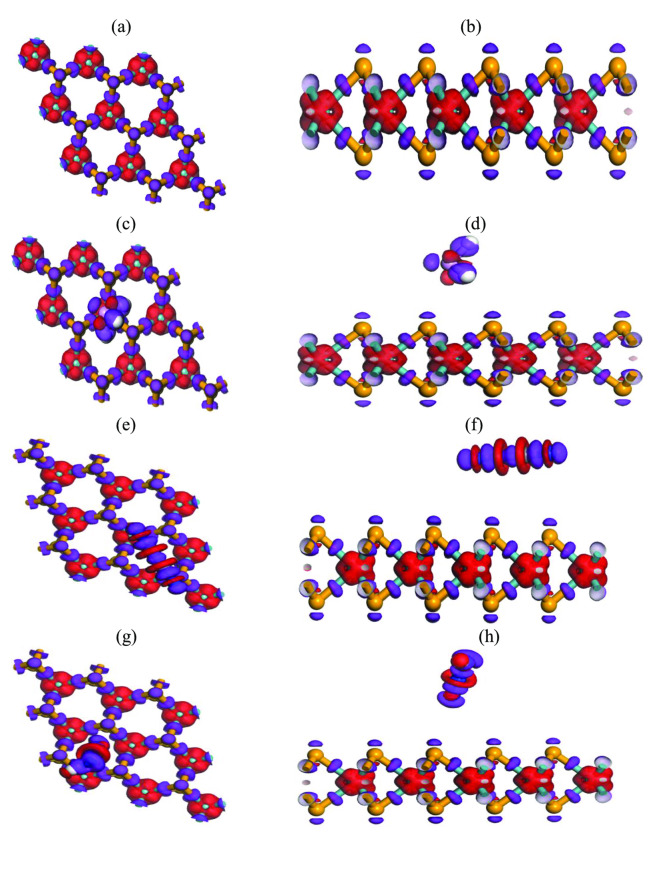
Electron density difference (EDD) for pristine (a) front and (b) edge view of MoSe_2_, (c) front and (d) edge view of MoSe_2_+PH_3_, (e) front and (f) edge view of MoSe_2_+C_2_N_2_, (g) front and (h) edge view of MoSe_2_+HN_3_.

**Figure 4:**
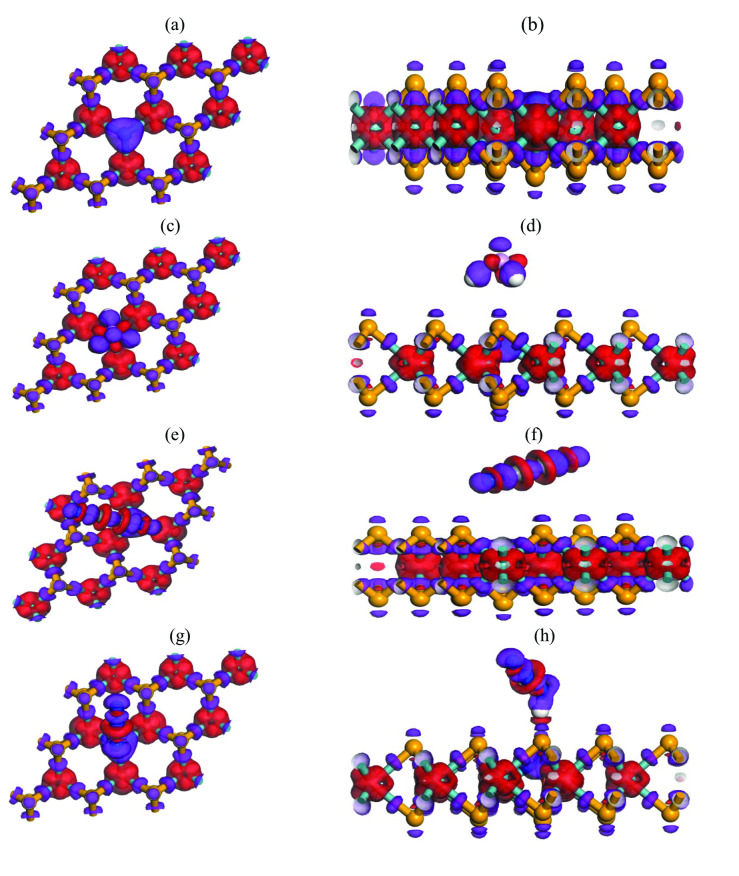
Electron density difference (EDD) for doped (a) front and (b) edge view of N‐MoSe_2_, (c) front and (d) edge view of N‐MoSe_2_+PH_3_, (e) front and (f) edge view of N‐MoSe_2_+C_2_N_2_, (g) front and (h) edge view of N‐MoSe_2_+HN_3_.

The electron density difference (EDD) before and after gas adsorption in MoSe_2_ and N‐MoSe_2_ monolayers are illustrated in Figures 3 and 4, respectively. The red color indicates the positive charge density i. e., electron deficit region and the purple color indicates the negative charge density i. e., electron‐rich region. The EDD exhibits that for all the adsorbents, the electrons are transferred from the Mo to the Se and N atoms. The density of electrons close to the N atom is higher than the Se atom due to the high electronegativity of the N atom. In the complex structures, the electrons are transferred from both adsorbents to the selective toxic gases.

#### 3.3.3 Band Structure Analysis

**Figure 5:**
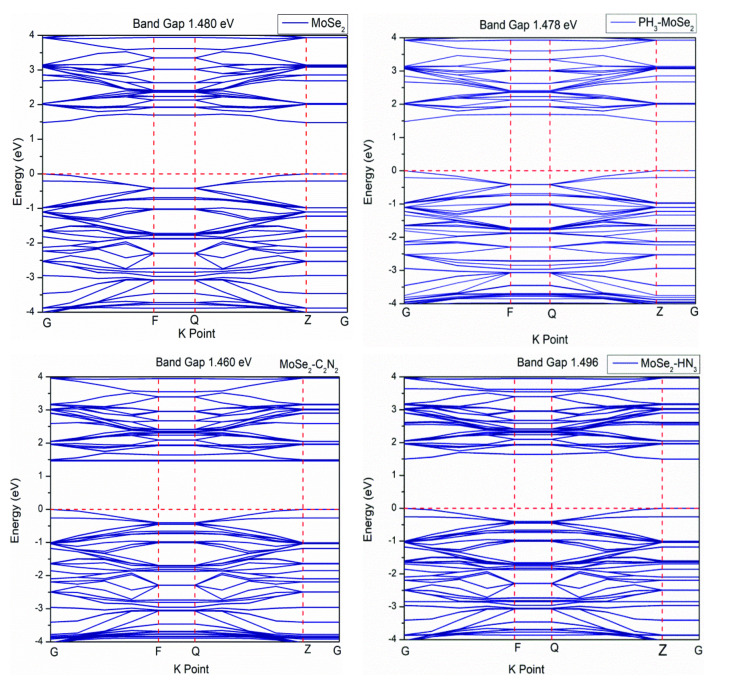
Band structures of before and after gas molecules adsorption on pristine MoSe_2_.

The band structures of MoSe_2_ (before and after gas adsorption) are represented in Figure 5. All the structures show a direct band gap with the bottom of the conduction band (BCB) and the top of the valence band (TVB) at ′*Z*′ K‐point. The energy band gap of MoSe_2_ is approximately 1.48 eV which is analogous to previous results.^[37]^ The band gap of the MoSe_2_ suffers slight variation due to gas adsorption.

**Figure 6:**
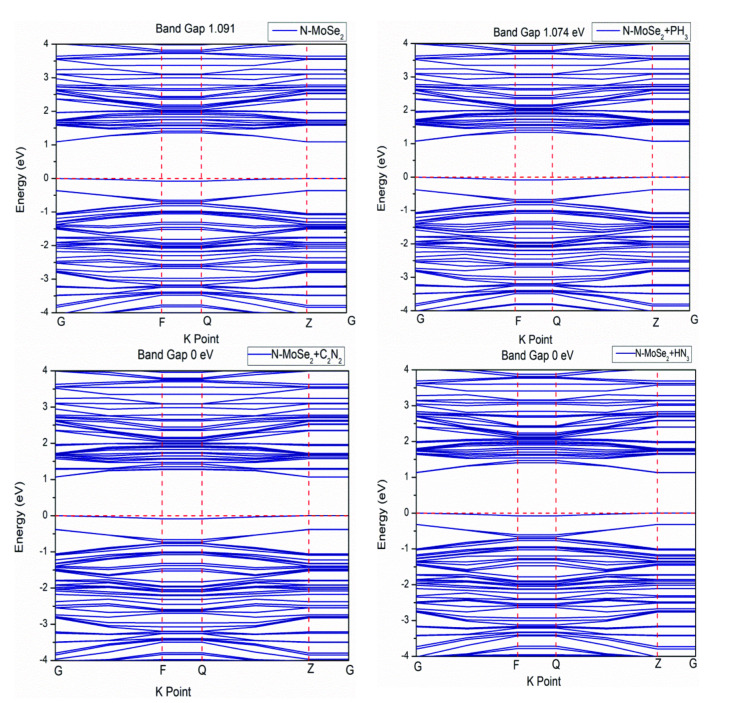
Band structures of before and after gas molecules adsorption on N‐MoSe_2_.

Figure 6 shows the band structure of N‐MoSe_2_ before and after toxic gas adsorption. The band gap decreases significantly due to N‐doping. N‐MoSe_2_ also possesses a direct band gap with BCB and TVB at ′Z′ K‐point. N‐MoSe_2_ exhibits metallic behavior with a zero band gap following the adsorption of C_2_N_2_ and HN_3_, while the band gap of N‐MoSe_2_ decreases slightly following the adsorption of PH_3_ gas.

#### 3.3.4 Density of States (DOS)

**Figure 7:**
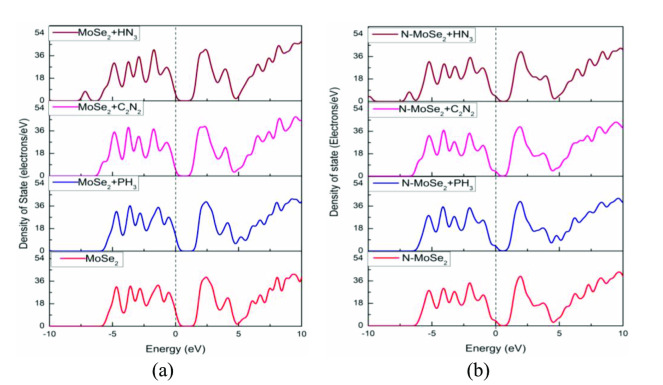
Total density of states (TDOS) of (a) before and after selective toxic gases adsorption on MoSe_2_ monolayer, (b) before and after selective toxic gases adsorption on N‐MoSe_2_ monolayers.

The density of states (DOS) is the number of electronic states per unit volume per unit energy. To identify whether a material is an insulator, semiconductor or metal, DOS analysis is the most fundamental method. If there are no energy states present in the fermi level (E_F_), then it indicates the presence of a bandgap which means that the material is either a semiconductor or an insulator.^[38]^ And if there is a continuous number of energy states present in E_F_, then it indicates the absence of bandgap which means that the material shows metallic behavior. Figure 7 shows the total density of states (TDOS) for MoSe_2_ and N‐MoSe_2_ monolayers before and after selective gas adsorption.

The TDOS close to the fermi level slightly increased because of N doping which resulted in a decrease in the band gap of MoSe_2_ after N doping. After gas adsorption in MoSe_2_ and N‐MoSe_2_, the valance band is closed to the E_F_ which indicates a decrease in the band gap. Small variations in the TDOS spectra of both MoSe_2_ and N‐MoSe_2_ monolayers before and after the adsorption of gas molecules are observed. But in the N‐MoSe_2_, there is a continuous number of states at E_F_, suggesting metallic behavior, following the adsorption of C_2_N_2_ and HN_3_ gas molecules. The presence of the continuity of TDOS also suggests that it is a good conductor of the electron. The TDOS of all structures satisfies their band structure.

**Figure 8:**
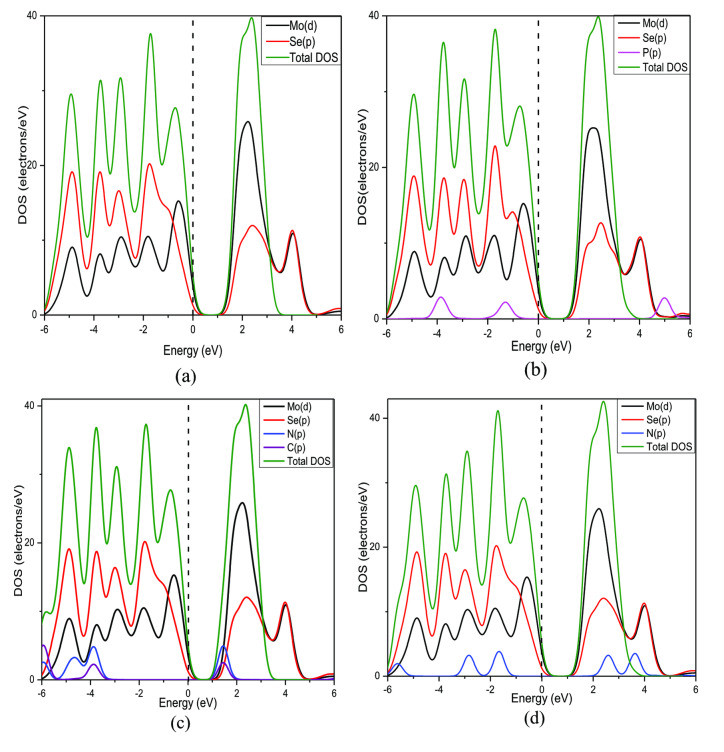
Partial Density of States (PDOS) of (a) Pristine MoSe_2_, (b) MoSe_2_+PH_3_, (c) MoSe_2_+C_2_N_2_, and (d) MoSe_2_+HN_3_.

**Figure 9:**
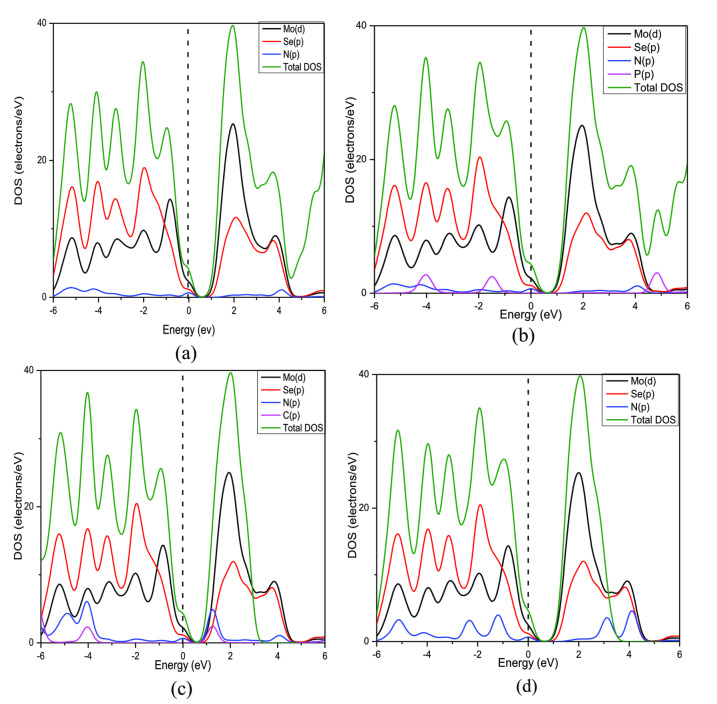
Partial Density of States (PDOS) of (a) N‐MoSe_2_, (b) N‐MoSe_2_+PH_3_, (c) N‐MoSe_2_+C_2_N_2_, and (d) N‐MoSe_2_+HN_3._

Figure 8 shows the partial density of states (PDOS) of MoSe_2_ before and after gas adsorption. Analyzing PDOS, it appears the 4 *d*‐orbital of Mo has the most contribution in the TVB and BCB which verifies the previous results.[^39^, ^40^, ^41^] It is also similar after selective gas molecules are adsorbed with a slight decrease in the energy gap. Figure 9 shows the PDOS of N‐MoSe_2_ before and after gas adsorption. Similar contributions of orbitals are observed in the PDOS of N‐MoSe_2_. However, a significant contribution of N's 2*p*‐orbital is observed in the BCB after C_2_N_2_ gas adsorption.

#### 3.3.5 Electrical Conductivity and Sensitivity Analysis

Electrical conductivity (σ ) of the MoSe_2_ and N‐MoSe_2_ monolayer before and after gas adsorption are compared in order to assess the sensitivity of the sensors. The following formula can be used to find the electrical conductivity of a material based on its temperature and bandgap.^[42]^

(4) $$\sigma =C\;e^{\left(\frac{-E_{g}}{2KT}\right)}$$

Here, C is a certain constant, T indicates the temperature (300 K); K is the Boltzmann's constant, and E_g_ is the band gap of the system. Table 9 shows the electrical conductivity of MoSe_2_ and N‐MoSe_2_ with selected toxic gases. Analyzing band structure, the band gap of the N‐MoSe_2_ monolayer after adsorption of C_2_N_2_ and HN_3_ gases is zero. Therefore, their electrical conductivity becomes maximum than other configurations. Electrical conductivity increases exponentially in response to a decrease in the bandgap. The highest electrical conductivity of the N‐MoSe_2_ monolayer after interacting with PH_3_ gas is because it has a lower band gap than other configurations. Also, pure N‐MoSe_2_ shows higher electrical conductivity than pristine MoSe_2_.

**Table 9:** Electrical conductivity (σ ) of MoSe_2_ and N‐MoSe_2_ before and after the selective gases adsorption.

Complex Structure	σ (×C Ω^−1^ m^−1^)
MoSe_2_	1.39× 10^−25^
N‐MoSe_2_	4.76× 10^−19^
MoSe_2_+PH_3_	1.50× 10^−25^
MoSe_2_+C_2_N_2_	3.02× 10^−25^
MoSe_2_+HN_3_	7.52× 10^−26^
N‐MoSe_2_+PH_3_	9.19× 10^−19^

Sensitivity (S) is related to electrical conductivity by the following equation^[43]^

(5) $$S=\;\left|\frac{\sigma_{monolayer+gas\;molecules-\sigma_{monolayer}}}{\sigma_{monolayer}}\right|\times 100\;\%$$

Where, $\sigma_{monolayer+gas\;molecules}$ and $\sigma_{monolayer}$ are the electrical conductivity of the gas‐adsorbed MoSe_2_ (or N‐MoSe_2_) monolayer and electrical conductivity of the isolated MoSe_2_ (or N‐MoSe_2_) monolayer.

**Figure 10:**
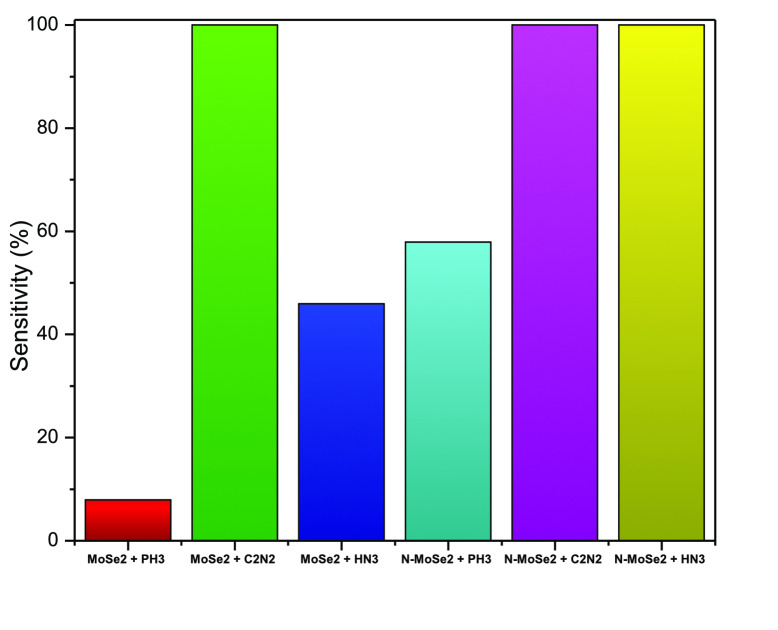
Sensitivity of selected gases with MoSe_2_ and N‐MoSe_2_ monolayer.

Figure 10 illustrates the bar chart of the sensitivity of MoSe_2_ and N‐MoSe_2_ monolayer toward PH_3_, C_2_N_2_, and HN_3_ gases at 300 K temperature. Here, we have taken the highest sensitivity to be 100 %. The MoSe_2_ monolayer shows the highest sensitivity (100 %) for C_2_N_2_ gas. On the other hand, N‐MoSe_2_ monolayer shows the maximum sensitivity for C_2_N_2_ and HN_3_ gases. Hence, the sensitivity of MoSe_2_ has significantly improved for the selected gases via N‐doping.

The MoSe_2_ showed significant differences in the sensitivity among the three gases which represent that MoSe_2_ is selective to these gases, i. e., MoSe_2_ can differentiate the sensing of PH_3_, C_2_N_2_, and HN_3_ gases. On the other hand, N‐MoSe_2_ is selective to PH_3_ gas; however, it cannot differentiate the adsorption of C_2_N_2_ and HN_3_ gases due to the same sensitivity for both gases.

#### 3.3.6 Work Function

The minimal amount of energy needed to carry an electron from the Fermi energy level to infinity is defined by the work function (Φ ), which is the energy difference between the vacuum and Fermi energy levels.^[18]^ We frequently used the Φ of a gas sensor, operating through the Kelvin method, to assess a substance's sensitivity.^[44]^ A Kelvin oscillator tool is used to calculate the Φ of a sensing material before and after gas exposure.^[45]^ The variation of Φ during the gas exposure operation reflects the sensitivity of the sensing material. An electrical device can detect a molecule through changes in its monolayer Φ upon adsorption; the greater the change, the simpler the detection process.^[46]^ The work function (Φ ) can be obtained by the following equation,^[46]^

(6) $$\Phi =E_{V}-E_{F}$$

where, E_V_ is the potential energy in the vacuum region and E_F_ is the Fermi energy.

The work functions (Φ ) and changes in the work function (ΔΦ ) of pristine and doped MoSe_2_ monolayer before and after the selective gases adsorption are depicted in Table 10. In MoSe_2_, following PH_3_ gas adsorption, the Φ increases dramatically, but following C_2_N_2_ and HN_3_ gas adsorption, it decreases. Also, the corresponding changes in ΔΦ is maximum after adsorbing PH_3_ gas. After PH_3_ and HN_3_ gas adsorption the Φ in N‐MoSe_2_ is greatly enhanced; however, following C_2_N_2_ gas adsorption, it is reduced. The greater changes of Φ are observed after adsorbing HN_3_ gas in N‐MoSe_2_. Since the variation in Φ is different for every gas adsorption both adsorbents can identify the adsorbed gas type in Φ ‐based gas sensor.

**Table 10:** Work function (Φ ) and changes in the work function (ΔΦ ) of MoSe_2_ and N‐MoSe_2_ before and after the selective gases adsorption.

Structure	Φ (eV)	ΔΦ (eV)
MoSe_2_	5.23	–
MoSe_2_+PH_3_	5.53	0.30
MoSe_2_+C_2_N_2_	5.43	0.20
MoSe_2_+HN_3_	5.21	0.02
N‐MoSe_2_	5.04	–
N‐MoSe_2_+PH_3_	5.12	0.08
N‐MoSe_2_+C_2_N_2_	4.59	0.14
N‐MoSe_2_+HN_3_	5.18	0.45

### 3.4 Optical Properties Analysis

**Figure 11:**
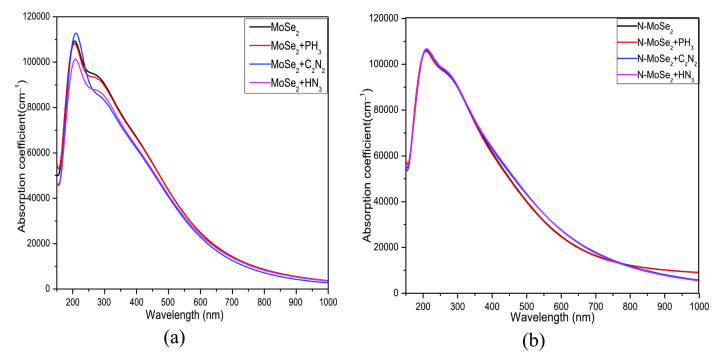
Absorption coefficient for (a) MoSe_2_, and (b) N‐MoSe_2_ monolayers with toxic gases.

Figure 11 shows the absorption coefficient (AC) for MoSe_2_ and N‐MoSe_2_ before and after gas adsorption. The AC pattern shows a slight peak shifting after selective gas adsorption on both adsorbents with the highest peak in the UV region. The maximum AC is in 10^5^ cm^−1^ order for all structures, which demonstrates that all the complexes absorb UV wavelength very strongly. Then if wavelength is increased, the AC is decreased continuously. The high AC make the MoSe_2_ and N‐MoSe_2_ potential materials for various optoelectronic applications. Very slight changes in optical absorption are observed due to gas adsorption.

**Figure 12:**
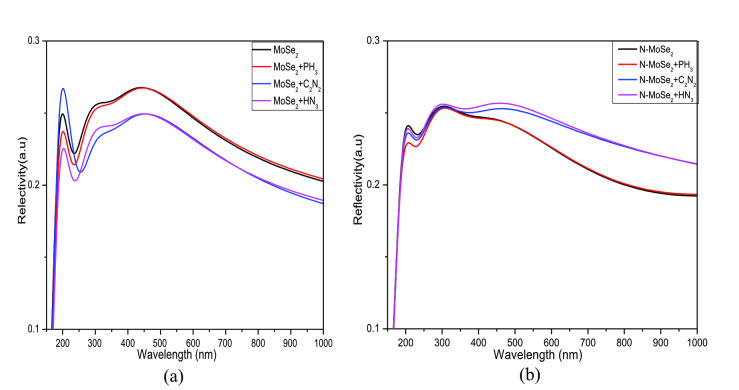
Reflectivity for (a) MoSe_2_ and (b) N‐MoSe_2_ monolayers with toxic gases.

Reflectivity is the ability of a surface to reflect incident photon energy. The optical reflectivity of MoSe_2_ and N‐MoSe_2_ before and after the adsorption of selective gases is shown in Figure 12. Both MoSe_2_ and N‐MoSe_2_ reflectivity changed significantly after gas adsorption. The reflectivity of MoSe_2_ decreased in the visible region after gas adsorption whereas in the case of N‐MoSe_2_, the opposite trend is observed.

**Figure 13:**
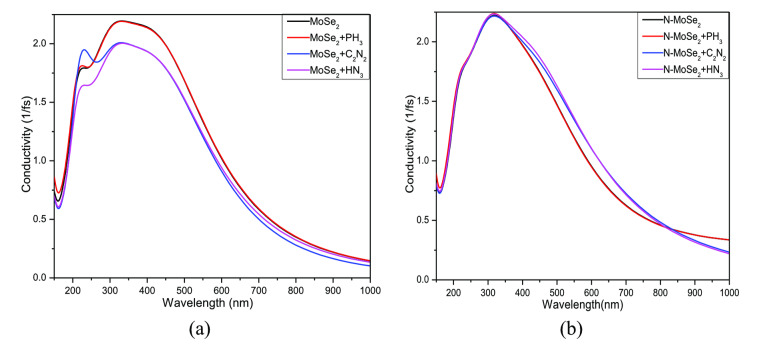
Conductivity for (a) MoSe_2_ and (b) N‐MoSe_2_ monolayers with toxic gases.

Figure 13 shows the optical conductivity (OC) of MoSe_2_ and N‐MoSe_2_ before and after toxic gas adsorption. The OC of MoSe_2_ falls in the visible region after gas adsorption. No significant peak shifting is observed due to gas adsorption. Here, conductivity is high after adsorbed C_2_N_2_ and HN_3_ gas by N‐MoSe_2_ which satisfies the band structure property. Both adsorbents show high OC in the visible region which is suitable for various optoelectronic applications.

## 4 Conclusions

The adsorption of PH_3_, C_2_N_2_ and HN_3_ toxic gases on MoSe_2_ and N‐MoSe_2_ has been investigated using DFT calculation. To understand the stability and sensing behavior, we have also studied the geometrical, electronic and optical properties of those monolayers and complex structures. The values of cohesive energy of the MoSe_2_ and N‐MoSe_2_ monolayers are −5.216 eV and −5.333 eV indicating these monolayers are stable. The doping of the N atom in MoSe_2_ results in a remarkable change in adsorption energy and electronic properties. Adsorption energy analysis also displayed that HN_3_ gas in the N‐MoSe_2_ shows maximum adsorption energy of −0.198 eV. After N doping, the band gap decreased significantly resulting in a low band gap semiconductor. After adsorption of C_2_N_2_ and HN_3_ on the surface of N‐MoSe_2_ monolayer, the band gap of the complexes reduces to 0 eV i. e. Semiconductor‐to‐metal transitions occurred. So band structure also shows that N‐MoSe_2_ revealed strong sensitivity for selective toxic gases compared to pristine MoSe_2_. The adsorption coefficient is significantly high in the UV energy region for all the adsorbents. Both adsorbents revealed high optical conductivity which suffers slight alteration due to gas adsorption. The optical properties make both adsorbents suitable for numerous optoelectronic applications. The high adsorption energy and variation in electronic properties ensure that N doping can enhance the sensitivity of MoSe_2_ toward selected toxic gases making N‐MoSe_2_ a superior candidate for gas sensing application. Both adsorbents can provide selective gas sensing performance in Φ ‐based gas sensors.

## Conflict of Interests

The authors declare no conflict of interest.

5

## Data Availability

The data that support the findings of this study are available in the supplementary material of this article.
